# Gene methylation profiles of normal mucosa, and benign and malignant colorectal tumors identify early onset markers

**DOI:** 10.1186/1476-4598-7-94

**Published:** 2008-12-31

**Authors:** Terje Ahlquist, Guro E Lind, Vera L Costa, Gunn I Meling, Morten Vatn, Geir S Hoff, Torleiv O Rognum, Rolf I Skotheim, Espen Thiis-Evensen, Ragnhild A Lothe

**Affiliations:** 1 Department of Cancer Prevention, Institute for Cancer Research, Norwegian Radium Hospital, Rikshospitalet University Hospital, Oslo, Norway; 2 Centre for Cancer Biomedicine, University of Oslo, Norway; 3 Department of Genetics, Portuguese Oncology Institute, Porto, Portugal; 4 Institute of Forensic Medicine, Rikshospitalet Medical Centre, University of Oslo, Oslo, Norway; 5 Faculty of Medicine, University of Oslo, Norway; 6 Medical Department, Rikshospitalet Medical Centre, Oslo, Norway; 7 Department of Medicine, Division of Gastroenterology, Telemark Hospital, Skien, Norway; 8 Department of Molecular Biosciences, University of Oslo, Oslo, Norway; 9 Surgical Department, Faculty Division Akershus University Hospital, University of Oslo, Oslo, Norway

## Abstract

**Background:**

Multiple epigenetic and genetic changes have been reported in colorectal tumors, but few of these have clinical impact. This study aims to pinpoint epigenetic markers that can discriminate between non-malignant and malignant tissue from the large bowel, i.e. markers with diagnostic potential.

The methylation status of eleven genes (*ADAMTS1*, *CDKN2A*, *CRABP1*, *HOXA9*, *MAL*, *MGMT*, *MLH1*, *NR3C1*, *PTEN*, *RUNX3*, and *SCGB3A1*) was determined in 154 tissue samples including normal mucosa, adenomas, and carcinomas of the colorectum. The gene-specific and widespread methylation status among the carcinomas was related to patient gender and age, and microsatellite instability status. Possible CIMP tumors were identified by comparing the methylation profile with microsatellite instability (MSI), *BRAF*-, *KRAS*-, and *TP53 *mutation status.

**Results:**

The mean number of methylated genes per sample was 0.4 in normal colon mucosa from tumor-free individuals, 1.2 in mucosa from cancerous bowels, 2.2 in adenomas, and 3.9 in carcinomas. Widespread methylation was found in both adenomas and carcinomas. The promoters of *ADAMTS1*, *MAL*, and *MGMT *were frequently methylated in benign samples as well as in malignant tumors, independent of microsatellite instability. In contrast, normal mucosa samples taken from bowels without tumor were rarely methylated for the same genes. Hypermethylated *CRABP1, MLH1*, *NR3C1*, *RUNX3*, and *SCGB3A1 *were shown to be identifiers of carcinomas with microsatellite instability. In agreement with the CIMP concept, MSI and mutated *BRAF *were associated with samples harboring hypermethylation of several target genes.

**Conclusion:**

Methylated *ADAMTS1*, *MGMT*, and *MAL *are suitable as markers for early tumor detection.

## Introduction

Most cases of colorectal cancer (CRC) originate from adenomas. The malignant potential of adenomas increases with size, grade of dysplasia, and degree of villous components,[1] along with the number and order of genetic and epigenetic aberrations.[2] The majority (~85%) of the sporadic carcinomas are characterized by chromosomal aberrations, referred to as a chromosomal unstable (CIN) phenotype, whereas the smaller group (~15%) typically show microsatellite instability (MSI) caused by defect DNA mismatch repair.[2] Most CIN tumors are microsatellite stable (MSS). A third molecular phenotype characteristic to a subgroup of CRC is the CpG island methylator phenotype (CIMP).[3] CIMP-positive tumors display methylation of multiple loci, are associated with proximal location in the colon, and are often microsatellite unstable. *BRAF *mutations are restricted to CIMP positive tumors, which may be sub-classified according to a certain combination of epigenetic and genetic changes.[4]

Here we have compared the time of occurrence and co-variation of multiple epigenetic markers in normal colon samples with those of adenomas and carcinomas in order to pinpoint early onset markers for neoplastic transformation.

## Materials and methods

### Tissue samples

Included in the present study are twenty-one normal colon mucosa samples from twenty deceased, cancer-free individuals, median age 52.5, range 33–86 (called N1 henceforth); 18 normal colon mucosa samples (N2) from 18 CRC patients, median age 70.5, range 24–89 (taken at distance (>10 cm) from the carcinoma); 63 adenomas, median size 8 mm, range 5–50 mm, from 52 individuals, median age 67, range 62–72; and 52 carcinomas from 51 patients, median age 70, range 33–92. The colon, including the rectum, is divided into proximal and distal sections; the proximal, or right side, spans from coecum to two thirds of the way across transversum; the distal, or left side, comprises the last third of the transversum, sigmoideum, and the rectum. This division originates from the primitive digestive tract, where the right side corresponds from the midgut, while the left side corresponds to the hindgut. The number of proximal *versus *distal samples in the series is as follows: N1 (10 *vs*. 11); N2 (7 *vs*. 11); adenomas (18 *vs*. 45); and carcinomas (17 *vs*. 35). The carcinomas included here are from a series evaluated to contain on average 84% tumor cells.[5] Nine of the N2 samples correspond to nine primary tumors analyzed here. Most of the normal colon samples (26/39) consisted of mucosa only, whereas the remaining ones were taken from the bowel wall. The adenomas were obtained from individuals attending a Norwegian colonoscopy screening program.[6] The carcinomas and the N2 samples are from a prospective series collected from 7 hospitals in the Oslo region of Norway.[5] The N1 samples were autopsy material collected by one of the authors.

The MSI status was determined by use of two mononucleotide markers, BAT25 and BAT26, and a panel of dinucleotide markers. Details regarding the assessment of MSI status are given in Additional file 1.

All samples belong to approved research biobanks and are part of research projects approved according to national guidelines (Biobank; registered at the Norwegian Institute of Public Health. Projects: Regional Ethics Committee and National Data Inspectorate).

### DNA methylation analyses

DNA from all samples was bisulfite modified and subjected to methylation specific polymerase chain reaction (MSP) for each gene.[7,8] Two of the authors independently scored all samples and the methylation status of all positive samples was confirmed by a second, independent round of MSP. If any discrepancies appeared, a third round of analysis was performed. In line with consensus scoring procedures, we considered carcinomas with band intensities as strong as the positive control (++) as methylated [see Additional file 2] for the gene promoter in question, while the benign lesions and normal mucosa were scored as positive also when weakly methylated, *i.e*. (+).

**Figure 1:**
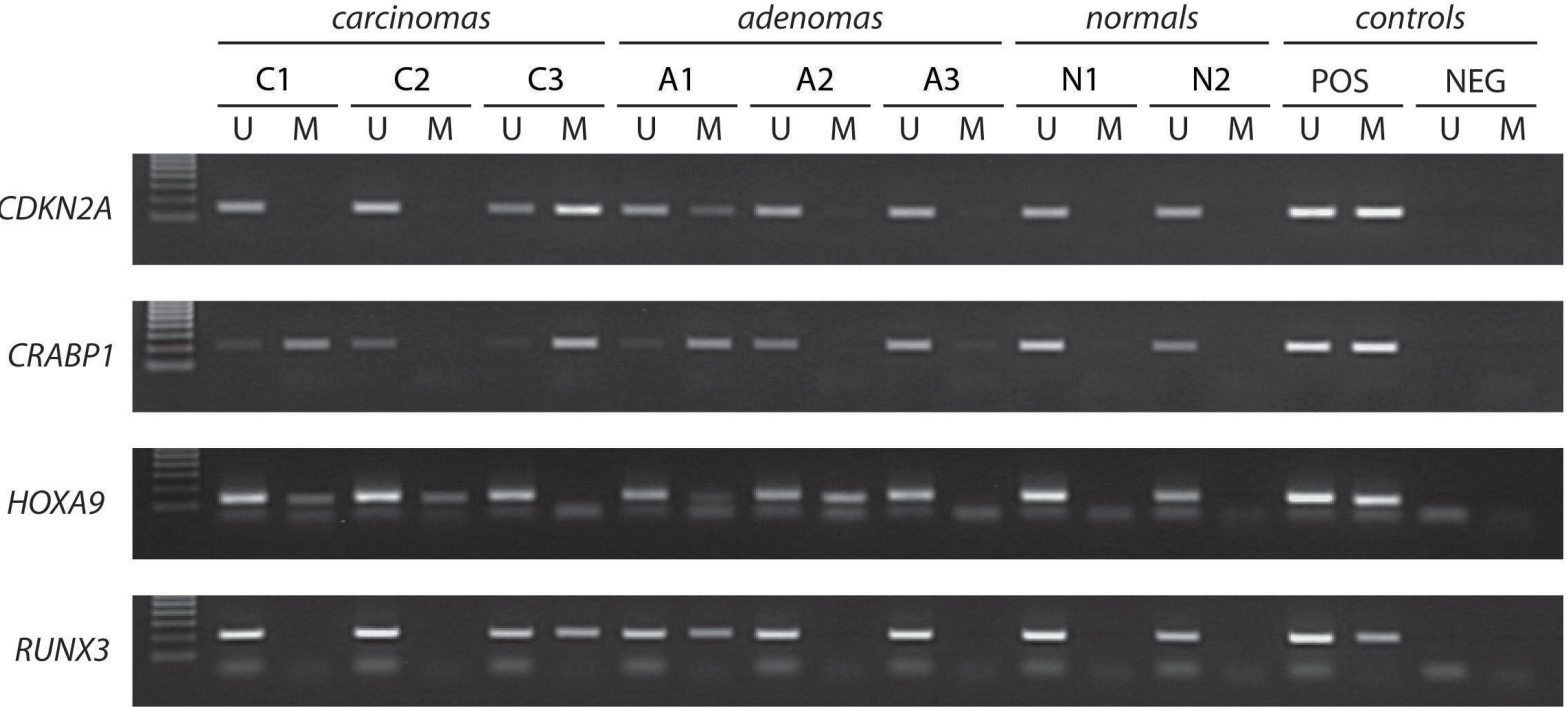
**Representative methylation results in colorectal tumors and normal mucosa**. Results of *CDKN2A*, *CRABP1*, *HOXA9*, and *RUNX3 *in selected samples are shown. Positive controls (POS): NB, normal blood, for the unmethylated reaction and IVD, *in vitro *methylated DNA, for methylated reaction. Negative controls: dH_2_O. U: unmethylated alleles, M: methylated alleles. The ladder (left lane) is the EZ Load™ 100 bp Molecular Ruler (BioRad, Hercules, CA, USA).

For detailed MSP protocol, primer sequences, and scoring criteria see Additional file 1. Representative MSP results can be seen in Figure 1.

Eleven genes, *ADAMTS1*, *CDKN2A *(encoding p16^INK4a^),*CRABP1*, *HOXA9*, *MAL*, *MGMT*, *MLH1*, *NR3C1*, *PTEN*, *RUNX3*, and *SCGB3A1 *(encoding HIN-1), were analyzed for promoter methylation by MSP. The methylation status of *ADAMTS1*, *CRABP1*, *MAL*, and *NR3C1 *for the present series,[9,10] and the methylation status of *CDKN2A*, *MGMT*, and *MLH1 *for the carcinomas [11] have previously been reported.

### Quantitative MSP

Primers and probes for quantitative MSP (qMSP) were designed to specifically amplify fully methylated bisulfite-converted complementary sequences of the promoter of interest. The primers and probe sequences used for the *MGMT *[GenBank: NM_002412] are listed in Additional file 3. To normalize for DNA input in each sample, a reference gene (*ACTB *[12]) was used.

Fluorescence based real-time PCR assays were carried out in a reaction volume of 20 μL, consisting of 16.6 mM ammonium sulphate; 67 mM trizma preset; 6.7 mM MgCl_2_; 10 mM mercaptoethanol; 0.1% DMSO; 200 μM each of dATP, dCTP, dGTP, and dTTP; 600 nM of each primer; 0.4 μL of Rox dye; 200 nM of probe; 1 unit of platinum Taq polymerase (Invitrogen, Carlsbad, CA, USA), and 2 μl of bisulfite-modified DNA as a template. PCR was performed in separate wells for each primer/probe set and each sample was run in triplicate. Additionally, multiple water blanks were used, and as positive and negative control we used commercial methylated and unmethylated DNA (Millipore, Temecula, CA, USA). A series of dilutions of methylated DNA after bisulfite conversion were used for constructing a standard curve to quantify the amount of fully methylated alleles in each reaction. All amplifications were carried out in 96-well plates on an 7000 Sequence Detection System (Applied Biosystems, Foster City, CA, USA), at 95°C for 2 min followed by 45 cycles of 95°C for 15 s, and 60°C for 1 min.

In order to adjust for the possible various amounts of bisulfite treated DNA input in each PCR, the qMSP levels were normalized against the respective values of the internal reference gene (*ACTB*). The ratio thus generated constitutes an index of the percentage of input copies of DNA that are fully methylated at the primer- and probe-binding sites. The ratio was multiplied by 100 for easier tabulation (methylation level = target gene/reference gene × 100).

A given sample was considered positive for promoter hypermethylation when amplification was detected in at least 2 of the triplicates of the respective qMSP analysis. The qMSP threshold was determined by adjusting the best fit of the slope and R2, using the calibration curve.

### Selection criteria for the 11 gene promoters analyzed in the present study

Some of the genes analyzed were known to be targeted through promoter methylation in cancer, including colorectal cancer (*SCGB3A1*, *RUNX3*, *CDKN2A*, *MLH1*, and *MGMT*). *HOXA9 *was a potential new methylation target in colorectal cancer. *ADAMTS1*, *CRABP1*, *MAL*, and *NR3C1 *were identified as novel epigenetically silenced target genes in colorectal cancer by our group.[9,10,13] They were selected to be tested in combination with known methylated genes in a large series of colorectal lesions to check for interdependencies. The methylation status of all included genes was compared in a series of normal mucosa from individuals without cancer with those of normal, benign and malignant tissue from the large bowel of cancer patients. Only two previous studies have compared gene methylation among the same four types of sample groups as investigated here.[14,15] The first only investigated one gene and the latter 10 genes among which only three overlapping the present selected gene list.

### Gene mutation status of *BRAF*, *KRAS *and *TP53*

The present carcinoma series form a part of a series previously studied for genetic changes, including *BRAF, KRAS *and *TP53*.[23,24] The specific mutation status of the individual tumors included here can be found in Additional file 4.

### Statistics

The 2 × 2 contingency tables were analyzed using Fisher's exact test and 3 × 2 tables were analyzed by the Pearson χ^2 ^test. Non-parametric analyses were performed using the Kruskal-Wallis and Mann-Whitney tests. An independent T-test was performed when comparing continuous normally distributed data with two groups. The bivariate correlation analysis was performed with Pearson's correlation. In order to determine age-specific methylation for the genes we used logistic regression analysis. All two-tailed *P*-values were derived from statistical tests using the SPSS15.0 software (SPSS, Chicago, IL, USA), and considered statistically significant at *P *≤ 0.05. The methylation heat-map was generated by average linkage hierarchical clustering and Pearson correlation distance measure, using the SpotFire DecisionSite^®^9.0 software.

Seven individuals had multiple polyps in the colon, and to exclude potential bias when analyzing patient data such as sex and age, one polyp from each individual was randomly selected for statistical analyses.

## Results

### MSI status of colorectal tumors

Two of sixty-three (3%) polyps displayed MSI. Both were large (≥10 mm) and located in the proximal colon. The carcinomas were pre-selected according to MSI-status and 27/52 (52%) were MSI-positive.

### DNA promoter methylation in normal mucosa, adenomas, and carcinomas

**Figure 2:**
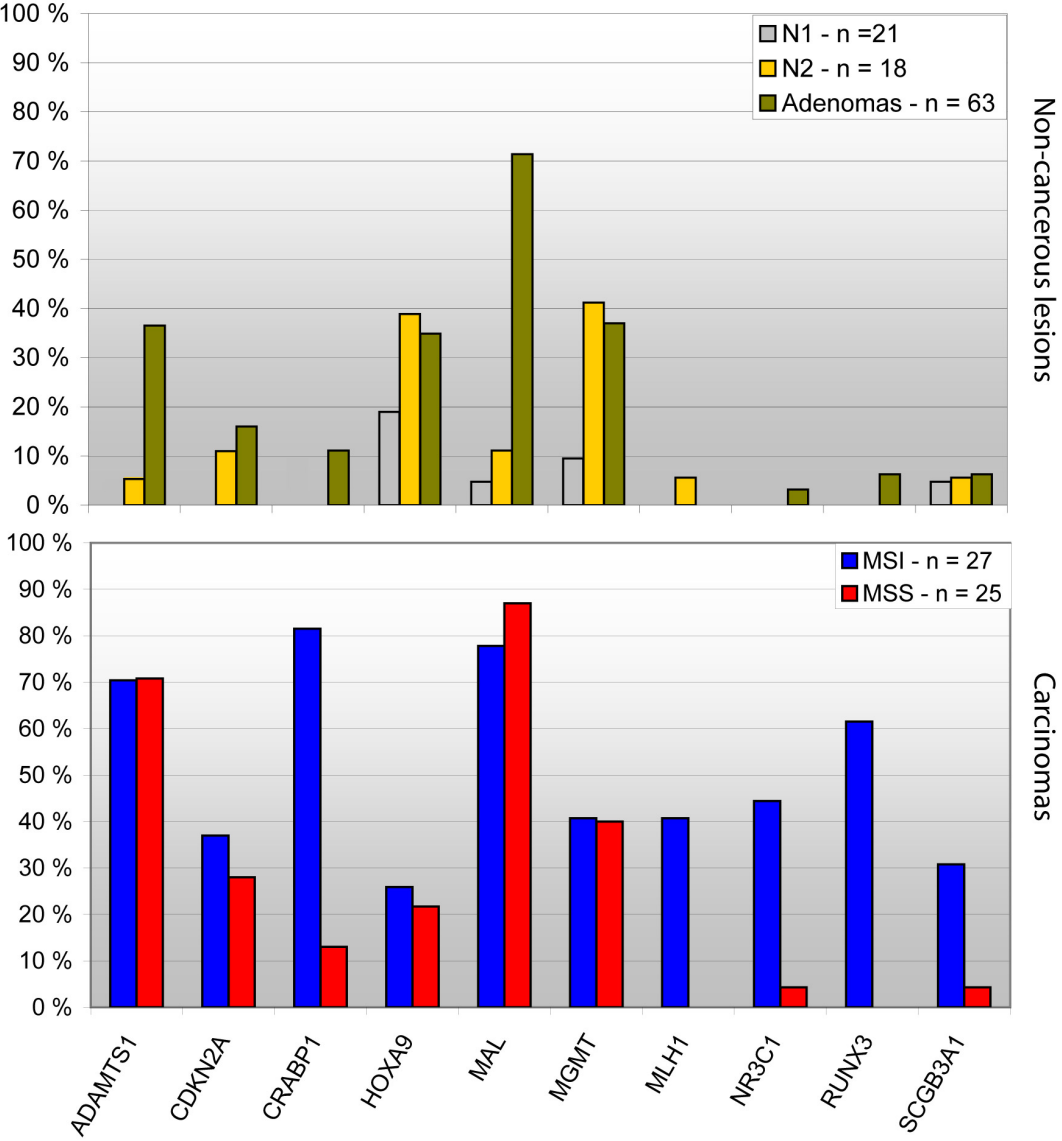
**Methylation profiles of normal mucosa, adenomas, and carcinomas**. Eleven genes were analyzed by MSP. Upper panel: non-cancerous lesions; lower panel: carcinomas stratified according to MSI-status. X-axis, the analyzed genes; Y-axis, the percentage of methylated samples. N1: normal colon samples from cancer-free individuals; N2: normal colon samples from cancer patients; MSI: microsatellite instability; MSS: microsatellite stability.

The results of the MSP analyses of all samples and each gene are summarized in Figure 2, Table 1, and Additional file 5. The mean number of genes methylated per sample was 0.4 for the N1 group, 1.2 for N2, 2.2 for adenomas, and 3.9 for carcinomas, and was significantly different among the groups using Kruskal-Wallis test; *P *< 0.0001 (mean rank N1, 10.2; N2, 17.3; adenomas, 23.1; and carcinomas, 31.4). Overall, 6/21 (29%) of the N1 samples, 9/18 (50%) of the N2 samples, 52/63 (83%) of the adenomas, and 48/52 (92%) of the carcinomas, were methylated in one or more of the eleven analyzed genes.

**Table 1:** Gene promoter methylation and microsatellite instability

	**ADAMTS1**		**CDKN2A**		**CRABP1**		**HOXA9**		**MAL**		**MGMT**		**MLH1**		**NR3C1**		**RUNX3**		**SCGB3A1**	
	**M**	**U**	**M**	**U**	**M**	**U**	**M**	**U**	**M**	**U**	**M**	**U**	**M**	**U**	**M**	**U**	**M**	**U**	**M**	**U**

**Sample type**																				
N1	**0**	**21**	**0**	**21**	**0**	**21**	**4**	**17**	**1**	**20**	**2**	**19**	**0**	**21**	**0**	**21**	**0**	**1**	**1**	**20**
N2	**1**	**17**	**2**	**16**	**0**	**18**	**7**	**11**	**2**	**16**	**7**	**10**	**1**	**17**	**0**	**18**	**0**	**1**	**1**	**17**
Adenoma	**23**	**40**	**10**	**53**	**7**	**53**	**22**	**40**	**45**	**18**	**23**	**39**	**0**	**63**	**2**	**61**	**4**	**4**	**4**	**59**
Carcinomas	**36**	**15**	**17**	**35**	**25**	**25**	**12**	**38**	**41**	**9**	**21**	**31**	**11**	**41**	**13**	**37**	**16**	**9**	**9**	**40**
**MSI status**																				
**Carcinomas**																				
MSI	**19**	**8**	**10**	**17**	**22**	**5**	**7**	**20**	**21**	**6**	**11**	**16**	**11**	**16**	**12**	**15**	**16**	**8**	**8**	**18**
MSS	**17**	**7**	**7**	**18**	**3**	**20**	**5**	**18**	**20**	**2**	**10**	**15**	**0**	**25**	**1**	**22**	**0**	**1**	**1**	**22**
P value	**NS**		**NS**		**<0.0001**		**NS**		**NS**		**NS**		**<0.0001**		**0.001**		**<0.0001**		**0.026**	

Abbreviations: M, methylated samples; U, unmethylated samples; NS, not significant; N1: non-cancerous normal samples; N2: normal samples from cancer patients. MSI status data is listed for the individual polyp. In some cases a patient may have several polyps.

Statistically significant differences in methylation frequencies among sample groups were also evident at the single gene level. *ADAMTS1*, *CDKN2A*, *CRABP1*, *MLH1*, *NR3C1*, *RUNX3*, and *SCGB3A1 *showed increasing methylation frequencies from adenomas to carcinomas, while *HOXA9*, *MAL*, and *MGMT *displayed overall equal methylation frequencies in all tumor subgroups. *PTEN *was unmethylated in carcinomas, and was thus not investigated in adenomas or included in the figures, tables (except Additional file 4) or statistics.

The more frequent promoter hypermethylation found among N2 samples compared with N1 samples was apparent both for the total number of methylated genes, and at the individual gene level (*MGMT*, *P *= 0.055). The reliability of our MSP scorings was tested by quantitative MSP analysis of one example gene performed in a blinded manner in another lab. The results were in perfect concordance [see Additional file 6].

No difference was seen in methylation frequencies between N2 samples with corresponding MSI-positive carcinomas (n = 6) and those with corresponding MSS carcinomas (n = 12).

Overall, gene methylation frequencies were higher among MSI than among MSS carcinomas, and were statistically significant for *CRABP1*, *MLH1*, *NR3C1*, *RUNX3*, and *SCGB3A1 *(*P *≤ 0.0001, *P *≤ 0.0001, *P *= 0.001, *P *≤ 0.0001, and *P *= 0.03, respectively). Methylation of these genes showed a strong association to proximal carcinoma location, demonstrating the close connection between high methylation levels, proximal location and MSI (*P *≤0.0001, *P *≤ 0.0001, *P *≤ 0.001, *P *= 0.001, and *P *= 0.04, respectively). Association to site was also seen for *HOXA9 *in N1 samples (*P *= 0.04). *HOXA9 *was also more frequently methylated among non-cancerous normal mucosa (n = 20) from older patients compared to younger patients, indicating age-specific methylation (*P *= 0.025). However, this was not confirmed among the larger group of carcinomas (n = 52).

### Interdependence among hypermethylated genes

From bivariate correlation analysis [see Additional file 7], methylation of *MLH1 *was correlated with methylation of *CRABP1 *(correlation coefficient 0.51; *P *= 5 × 10^-11^), *NR3C1 *(correlation coefficient 0.72; *P *= 1 × 10^-25^) and *RUNX3 *(correlation coefficient 0.57; *P *= 6 × 10^-14^). Methylation of *RUNX3 *itself was strongly correlated to methylation of both *NR3C1 *(correlation coefficient 0.75; *P *= 5 × 10^-28^) and *CRABP1 *(correlation coefficient 0.67; *P *= 3 × 10^-20^). Methylation of *NR3C1 *and *CRABP1 *was also correlated (correlation coefficient 0.59; *P *= 4 × 10^-15^), as well as *ADAMTS1 *and *MAL *(correlation coefficient 0.53; *P *= 2 × 10^-12^).

**Figure 3:**
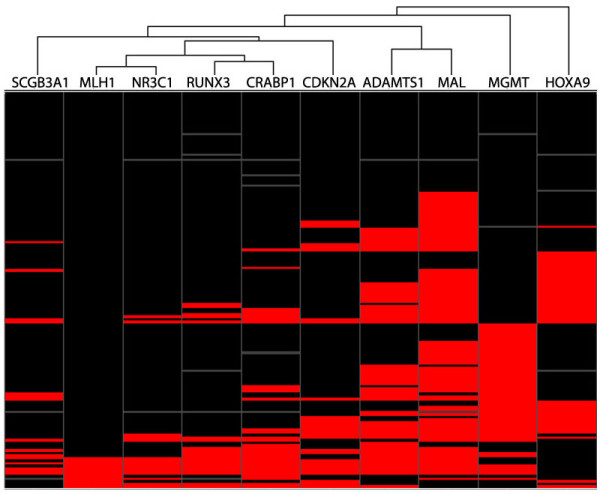
**Methylation HeatMap**. Hierarchical clustering reveals that methylation of *NR3C1 *and *RUNX3 *are most closely related, followed by *MLH1 *and *CRABP1*. Methylation of *MGMT *and *HOXA9 *are most independent both from each other and from rest of the set. The genes are presented in columns, while the samples are presented in rows. Black, unmethylated; red, methylated; and grey, missing values.

Hierarchical clustering of samples according to gene methylation status showed that *MLH1 *and *NR3C1 *were most closely related, followed by *RUNX3 *and *CRABP1*. In contrast, *HOXA9 *and *MGMT *displayed methylation patterns independent from each other and the other genes (Figure 3).

### Widespread methylation

**Figure 4:**
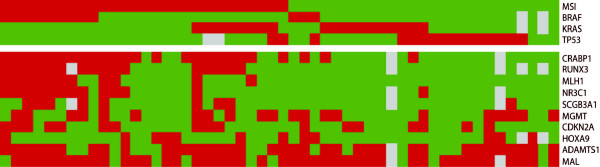
**Genetic and epigenetic changes in colorectal carcinomas with known microsatellite status**. The results are visualized according to genetic (top part of the figure) and epigenetic changes (lower part of the figure). The results are organized according to MSI, followed by *BRAF*-, *KRAS*-, *TP53*- and methylation-status of the MSI associated genes.

Several samples harbored simultaneous promoter methylation of two or more of the analyzed genes [see Additional file 8]. The distribution of methylated gene numbers per sample did not appear to be bimodal. Neither N1 nor N2 samples displayed methylation of five or more genes, here denoted widespread methylation. Seven of 63 (11%) adenomas displayed widespread methylation, and these were by far larger in size (mean = 19 mm) than the remaining adenomas (mean = 10 mm; *P *= 0.013). In carcinomas, widespread methylation was seen more frequently in MSI (16/27; 59%) than in MSS (3/25; 12%) samples (*P *= 0.001). All sixteen MSI samples with widespread methylation showed similar molecular profiles when DNA methylation status, *TP53*-, *KRAS*-, and *BRAF*-mutation status were considered, in line with a CIMP positive phenotype (Figure 4). The three MSS samples with widespread methylation included one tumor with *TP53 *mutation, one with both *TP53 *and *KRAS *mutation and one with *BRAF *mutation.

**Figure 5:**
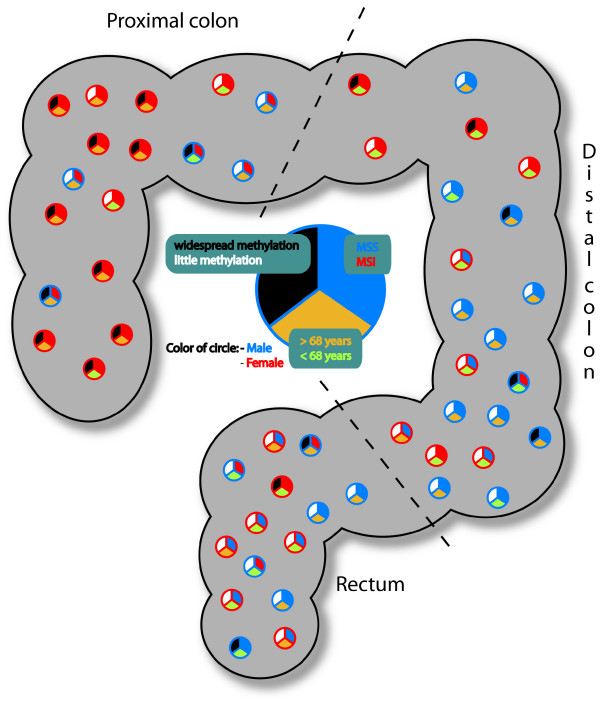
**Distribution of colorectal carcinomas according to site associates with sex, age, MSI-status and methylation frequencies**. The circles indicate 52 carcinomas placed according to site, the red circle = female, the blue = male. Top right section of the circle: blue = MSS, red = MSI. The lowest section: green = patient <68 years of age, yellow = patient ≥ 68 years. Widespread methylation is given in the top left section: white = methylation in < 5 genes, black = widespread methylation ≥ 5 genes.

The distribution of the carcinomas combined with information regarding sex, age, MSI-status, and widespread methylation is illustrated in Figure 5. From the figure we see that widespread methylation is associated with proximal tumors derived from elderly women.

## Discussion

We demonstrate in the present study aberrant promoter methylation of several genes, at variable frequencies, in the stepwise development of colorectal tumors.

An association between hypermethylation and lack of expression has previously been shown for all genes analyzed in the present study.[8-10,25-28] Although multiple genes are methylated in a cancer, only some are functionally involved in tumorigenesis, [29-31] whereas others with unknown functional contribution still may serve as good biomarkers from a diagnostic perspective.

### Comparing methylation profiles of normal mucosa, adenomas and carcinomas of the large bowel

The identified methylation profiles of normal colorectal tissues, adenomas, and carcinomas demonstrated a stepwise increase in CpG island promoter methylation towards malignancy, indicating that their inactivation plays a role in the progression of the tumor. This was evident both for widespread methylation and at the single gene level (increasing frequencies of methylation from benign to malignant stages) with the exception of *HOXA9*, *MAL*, and *MGMT*. The lack of increase in methylation frequencies between non-malignant adenomas and carcinomas for these three genes may suggest that they are more important in the initiation of cancer, rather than in progression. These genes in addition to *ADAMTS1 *were also hypermethylated in comparable frequencies among MSS and MSI carcinomas. These observations, and the fact that the separation of the MSI- and MSS-pathway is thought to occur early in colorectal tumorigenesis suggest that alterations of the four genes represent early events. *ADAMTS1 *is believed to be an inhibitor of both angiogenesis and endothelial proliferation,[32] features commonly activated in cancer, as a tumor must turn on angiogenesis in order to grow larger than 1–2 mm^3^[33]. Members of the HOX gene family are shown to be commonly altered in several cancers, and to the best of our knowledge, this is the first report of *HOXA9 *methylation in colorectal neoplasms. *HOXA9 *methylation has received increasing interest in recent time as it is included in the HOXA-cluster which harbors methylation over a larger area than just a single promoter, indicating that methylation may mimic genetic micro-deletions and turn off a cluster of genes rather than just one at the time, i.e. yet another example of long range epigenetic silencing. [34-36]. *MAL *is involved in T-cell differentiation, especially in the late or intermediate stages.[37] It is also involved in polarization of epithelial cells caused by apical transport of lipids and proteins. Loss of cell polarity is often seen in neoplastic transformation.[38] For *MGMT *the early involvement is further supported by the fact that promoter methylation has previously been identified in aberrant crypt foci.[39]

Our data do not suggest that any of the markers included here were methylated in an age dependent manner. Of the 11 analyzed genes, six were unmethylated in all normal samples from non-affected individuals, excluding them as age-specific methylation targets. For two genes (*SCGB3A1 *and *MAL*) only one of 21 samples was methylated. Although the sample in question was from an older individual (75 years), the resulting overall methylation frequency was only 5%. This is in strong contrast to the frequent reported age-specific methylation of the *N33 *gene, which shows approximately 46% methylation among normal samples in general and 58% methylation in normal samples from individuals over 60 years.[40]*HOXA9 *is the only gene in the present study harboring "frequent" promoter methylation in normal samples (19% overall, and 43% for individuals of 60 years or older). Binary regression analysis resulted in a significant *P *value, however, when using the same statistical analysis in the tumor sample series age dependence could not be confirmed. Both technical and biological aspects influence the interpretation of DNA promoter methylation analyses.

The importance of primer design is emphasized in the *PTEN *assay. Promoter hypermethylation of *PTEN *has been frequently reported in various tumor types, including CRC. [16-19] However, the majority of MSP primer sets used have failed to discriminate between *PTEN *and its frequently methylated pseudogene, leading to a high rate of false positives.[20] In the present study, we used MSP primers specifically designed to amplify the protein-encoding *PTEN *gene,[21] and showed that *PTEN *was not subject to promoter hypermethylation in colorectal carcinomas. A novel study confirms that methylation of *PTEN *is an unusual event in colorectal cancer as a whole.[22]

### Interdependence among hypermethylated genes and widespread methylation

The hierarchical clustering analysis of gene promoter methylation status in normal, benign, and malignant samples confirmed that the distribution of *HOXA9 *and *MGMT *methylation frequencies across sample groups differed from the other genes. Overall, methylation of *NR3C1 *and *RUNX3 *had the highest correlation (Figure 3 and Additional file 7), in addition to *MLH1*, which was also closely related to *NR3C1 *and *RUNX3*. Furthermore, the present study confirmed that hypermethylation of *MLH1 *was characteristic of right-sided sporadic colon tumors with MSI.[41] The lack of *MLH1 *hypermethylation in adenomas analyzed in the present study supports the theory that CIMP and MSI-tumors arise from sessile serrated polyps rather than from adenomas.[42]*NR3C1*, *RUNX3*, *CRABP1*, and *SCGB3A1 *were also shown to have the same characteristics as *MLH1*, supporting the hypothesis that DNA methylation plays a more prominent role in proximal than in distal carcinogenesis. *CRABP1*, *MLH1*, *NR3C1*, and *RUNX3 *have recently been shown to belong to a panel of epigenetically regulated genes which best discriminate between CIMP-positive and CIMP-negative tumors, a phenotype strongly related with MSI status.[43]

We found that the MSI positive samples with V600E *BRAF *mutations were accompanied by promoter hypermethylation of several genes, in agreement with the CIMP phenotype (Figure 4). Furthermore, we also confirmed that MSS tumors with *TP53 *mutations had less overall methylation, and thus in agreement with a CIMP negative phenotype. *KRAS *mutations were evenly distributed between MSI and MSS samples but seemingly the KRAS/MSI samples had more methylation than KRAS/MSS samples. Interestingly, three MSS samples had *BRAF *mutations, and all differed from the V600E mutation found among the MSI tumors.

### Methylation markers suitable for early tumor detection

For genes previously analyzed for promoter methylation in normal colon samples, our results are within the expected range (*CDKN2A*, 0–33% (range of samples 9–100, total methylation frequency ~4%) [44-57]; *MGMT*, 0–39% (range of samples 12–220, total methylation frequency ~7%)[14,15,44,49,50,53,56-61]; and *MLH1*, 0–50% (range of samples 8–100, total methylation frequency ~5%)).[44,46,49,50,52,53,55-57,62-67]*SCGB3A1 *and *RUNX3 *have previously been analyzed in only one study, and both were unmethylated in 57 normal samples.[48] The study showing the highest methylation frequency of *CDKN2A *and *MLH1 *were biased towards normal samples taken distant from MSI- and CIMP-positive tumors,[46] thus a higher degree of methylation might be expected.

A suitable, highly specific, biomarker should be unmethylated in normal mucosa from healthy individuals and frequently methylated in carcinomas, and possibly also in benign lesions. To date, only few such markers have been identified,[10,68,69] and one of the most suitable ones, Vimentin, is non-expressed in a normal, healthy, colon.[69] The fact that an important biomarker is non-expressed in normal tissue supports the choice of a low threshold for methylation positive early lesions, applied in the present search for early onset biomarkers. Hypermethylation of genes such as *ADAMTS1 *and *MAL *are also suitable biomarkers for early detection, as they are infrequently methylated in normal mucosa taken from individuals without cancer (0% and 5%, respectively), but highly methylated in malignant lesions (71% and 82%, respectively)[9,13]. In addition, both are frequently hypermethylated among the adenomas (37% and 71%, respectively) independent of size. Of course, sufficient sensitivity and specificity of these hypermethylation markers must be shown in feces or blood samples for the purpose of non-invasive testing. It should be note that this is an obstacle yet to be overcomed by suggested markers in existing non-invasive tests.

It has been speculated that methylation of specific genes, such as *MGMT*, may yield a so-called "field effect", providing favorable conditions for further alterations which eventually might lead to tumor formation.[58,70] The initial steps in tumorigenesis might be due to an epigenetic disruption of a progenitor/stem cell which may be followed by genetic mutations of gatekeeper genes, and the subsequent acquisition of other genetic and epigenetic alterations.[71] This model provides a possible explanation of why we see relatively high methylation frequencies for genes such as *MGMT*, and *HOXA9 *in normal samples taken from cancer patients.

Summarized, this study has shown that gene-specific promoter hypermethylation is an early event in colorectal tumorigenesis, exemplified by hypermethylation of *MGMT *in adenomas and normal mucosa from cancer patients, and by the high frequency of *ADAMTS1 *and *MAL *methylation in polyps irrespective of size. These markers are suitable as part of a panel aiming at detecting early colorectal lesions, and possibly a field effect in a "labile" colon. In general, we saw that aberrant CpG island hypermethylation increased with malignancy. Finally, methylation of *CRABP1*, *MLH1*, *NR3C1*, *RUNX3*, and *SCGB3A1 *were identifiers of MSI carcinomas.

## Competing interests

The authors declare that they have no competing interests.

## Authors' contributions

All authors have read and approved the final version of the manuscript. TA was main responsible for the laboratory analyses, performed statistical analyses, made all figures and drafted the manuscript. GEL participated in the study design, in experimental analyses and in the preparation of the manuscript. VLC performed the quantitative methylation specific PCR analysis. GIM collected the cancer series and provided the clinicopathological information. MV participated in the screening study from which we received adenomas and patient information. GSH was responsible for the screening study from which we received adenomas and patient information. TOR collected and provided normal mucosa from non-cancerous individuals, the carcinoma series and participated in scientific discussions. RIS contributed to the statistical analyses and in scientific discussions. ETE participated in the screening study from which we received adenomas and patient information as well as in study design and scientific discussions. RAL conceived the study, participated in the evaluation of the results and in manuscript preparation.

## Supplementary Material

Additional file 1 **Supplementary information.** Methodological details which are not crucial for the understanding of the work, as well as Additional figure legends. Click here for file

Additional file 2 **Titration of methylated DNA template illustrates the scoring thresholds for the methylation-specific polymerase chain reaction.** Determination of scoring thresholds is visualized by a titration series of the *RUNX3 *gene. Click here for file

Additional file 3 **PCR primers used for methylation-specific PCR and microsatellite instability analyses.** Information on primers and PCR details. Click here for file

Additional file 4 **Genetic and epigenetic raw data.** Raw data from all analyses are listed for each tumor included. Click here for file

Additional file 5 **Summarized methylation raw data.** Methylation frequencies are presented for each of the eleven analyzed genes in the 4 different sample types according to what methylation level they exhibited (strong, weak or no methylation). Click here for file

Additional file 6 **Comparison between MSP and quantitative MSP in normal mucosa samples.** Hypermethylation of was analyzed with both non-quantitative- and quantitative MSP. Here the results from each method are presented. Click here for file

Additional file 7 **Correlation between methylated genes.** A correlation table including all analyzed for promoter hypermethylation shown that genes commonly methylated in MSI tumors are highly correlated. Click here for file

Additional file 8 **Widespread methylation among normal colorectal samples, adenomas, and carcinomas.** A histogram showing the total number of methylated genes per sample in non-cancerous normal mucosa, normal mucosa taken in distance from a primary tumor, adenomas, and carcinomas stratified according to MSI status. Click here for file
