# Supplementary material for: Gene methylation profiles of normal mucosa, and benign and malignant colorectal tumors identify early onset markers
Source: Mol Cancer. 2008 Dec 31;7:94. doi: 10.1186/1476-4598-7-94 (PMC2639620; doi:10.1186/1476-4598-7-94)
Supplement: Additional file 1 — Supplementary information. Methodological details which are not crucial for the understanding of the work, as well as Additional figure legends. [file 1476-4598-7-94-S1.doc]

# Supplementary information

## Bisulfite modification and methylation specific polymerase chain reaction (MSP)

DNA from all samples was bisulfite modified, a chemical process in which unmethylated but not methylated cytosines are converted to uracil,[1,2] using either the CpGenome™ DNA modification kit (Serological, Norcross, GA, USA) or as previously described.[3]

**[4-13]**

## MSP protocol

For each gene, two primer pairs were used for MSP analysis, the first recognizes and anneals to methylated sequences only, whereas the second set amplifies unmethylated alleles. The unmethylated and methylated reactions of MSP were carried out in a total volume of 25µl containing 1xPCR Buffer (QIAGEN Inc., Valencia, CA), 200µM dNTP (Amersham Pharmacia Biotech Products Inc., Piscataway, NJ), 20pmol of each primer, and 0.625U HotStarTaq DNA Polymerase (QIAGEN). All primers were purchased from Medprobe AS (Oslo, Norway) and their sequences are listed in supplementary table 1 along with the magnesium concentration in each PCR, PCR program annealing temperature, and fragment size.

Human placental DNA (Sigma Chemical Co., St. Louis, MO) treated *in vitro* with *SssI* methyltransferase (New England Biolabs Inc., Beverly, MA) was used as a positive control for the methylated MSP, whereas DNA from whole blood was used as a control for the unmethylated reaction. Water was used as a negative PCR control in both reactions.

## Threshold for scoring methylated samples from MSP analysis

The band intensity of PCR products generated by the methylation-specific primer set varied considerably among samples analyzed, but was highly reproducible for each individual sample (data not shown). The results from the MSP analysis were visually scored according to three previously described sample categories, strongly methylated, weakly methylated and unmethylated.[14] In brief, samples with equal or stronger band intensity than the positive control in the methylation specific reaction were denoted strongly methylated (++). Samples with less intense bands than the positive control were categorized as weakly methylated, whereas samples with very weak band intensity and samples with no visible PCR product were regarded as unmethylated. Using the methylation specific reaction of *RUNX3* MSP and a titration of the amount of methylated template as an example, samples with 50 to 100% methylated alleles would classify as strongly methylated, samples with 10 to 40% methylated alleles would classify as weakly methylated alleles, and samples with 0 to 5% methylated alleles would classify as unmethylated (supplementary figure 1). However, this is expected to vary from gene to gene according to the efficiency of the PCR reaction.

In line with consensus scoring procedures, we have considered carcinomas with strong band intensities (++) as methylation positive for the gene promoter in question, while the benign lesions and normal mucosa are scored as positive also when weakly methylated (+ and ++). When trying to detect early tumorigenic changes it is important to be able to detect the minor, as well as the major, changes. Minor changes may be present in only a small fraction of the cells in the lesion. By scoring the normal samples and early lesions with a low threshold, we increase the likelihood of also identifying these minor changes.

Among the N2 samples, DNA promoter methylation of the *MGMT* gene was analyzed both with qualitative and quantitative MSP (blinded test, using a previously set threshold for the qMSP analysis). Full concordance was seen in the results from the two analyses and all samples scored as methylated using qMSP had previously also been scored as methylated using the qualitative MSP, which in turn supports our choice of scoring threshold for the latter.

## Assessment of MSI status

To determine the MSI status of the benign tumors, two markers located within mononucleotide repeats, BAT25 and BAT26 were analyzed in tumor samples. The combined analyses of these two markers, or even just BAT26, can detect more than 99% of tumors with MSI.[15] 50 ng DNA template was amplified in a 10 µl reaction volume consisting of 1xQIAGEN® PCR buffer containing 1.5mM MgCl_2_ (QIAGEN GmbH, Hilden, Germany), 1.5-4 pmol BAT25 primers (sense primer labeled with HEX in the 5’end), 1.5-4 pmol BAT26 primers (sense primer labeled with 6-FAM in the 5’ end; DNA Technology AS, Aarhus, Denmark), 2 pmol of each of the four dNTPs, and 0.4 units of HotStarTaq™ Polymerase (QIAGEN®). The PCR annealing temperature was 55°C and the program included 27 cycles. Primer sequences are listed in supplementary table 1.

1 µl PCR product was mixed with 0.5μl GeneScan®-500 [TAMRA] Size Standard (PE Biosystems) and 12μl de-ionized formamide (Eastman Kodak, Rochester, NY, USA), denatured and separated by 16 minutes capillary electrophoresis at 60ºC (ABI PRISM™310 Genetic Analyzer; PE Biosystems).

The results were independently scored by two individuals. A second round of analyses confirmed the results. For carcinomas, the MSI status was previously established by use of the two BAT markers as well as several dinucleotides.[16] For the present study, we selected 27 MSI and 25 MSS primary carcinomas from this sample series.

# Figure legends – Additional figures

**Additional file 2. Titration of methylated DNA template illustrates the scoring thresholds for the methylation-specific polymerase chain reaction.**

The methylation specific reaction of the *RUNX3* MSP using a titration series of bisulfite treated methylated control (*in vitro* methylated DNA; IVD) mixed with unmethylated control (normal blood; NB) is shown. Percentages denote the relative amount of methylated control compared to the unmethylated control. Samples with equal or stronger band intensity than the positive control (wells 10 - 12) were denoted strongly methylated (++), whereas samples with weaker band intensity than the positive control (wells 5 – 9) were categorized as weakly methylated (+). Samples with very weak band intensity (wells 2 - 4) and samples with no visible PCR product (well 1) were regarded as unmethylated. The ladder (left lane) is the EZ Load™ 100bp Molecular Ruler.

**Additional file 3. PCR primers used for methylation-specific PCR and microsatellite instability analyses.** Abbreviations: Frg-size bp.; Fragment size in base pairs, An. Temp; annealing temperature in degrees Celcius.

**Additional file 4. Genetic and epigenetic raw data.**

Detailed genetic and epigenetic raw data is given per sample. Abbreviations: wt: wild-type; NP: not performed; M: methylated; U: unmethylated. All mutation details are given according to the correct nomenclature.

**Additional file 5. Summarized methylation raw data.**

Methylation frequencies are presented for each of the eleven analysed genes in the 4 different sample types. –: unmethylated, +: weakly methylated, and ++: strongly methylated. Abbreviations: NP: not performed; N1: normal samples from cancer-free individuals; N2: normal samples from cancer patients. The thresholds for determining methylation status are based on the titration of methylated alleles presented in supplementary figure 2.

**Additional file 6. Comparison between MSP and quantitative MSP in normal mucosa samples.** Methylation status of the individual sample is indicated with colors. Green: unmethylated; Orange: weakly methylated; Red: methylated; White: not determined. In the qMSP-column the quantitated level of methylation is listed. Both weak and strong methylation is considered to be methylated among our benign and non-benign samples.

**Additional file 7. Correlation between methylated genes**

The correlation coefficient (-1 🡪 1) is listed for each combination in the upper panel, and the *P*-value for this is listed in the lower panel. Correlation coefficients above 0.50 are presented in bold. The number of samples included in the bivariate correlation analysis is 146-154.

**Additional file 8. Widespread methylation among normal colorectal samples, adenomas, and carcinomas.** Percentage of A) normal samples and adenomas, and B) carcinomas displaying methylation of zero to ten of the promoters analyzed in the present study. Carcinomas are stratified according to MSI status. Only samples with informative results in eight of ten genes analyzed are included. X-axis: Number of methylated genes; Y-axis: Frequency of samples. N1: normal samples from cancer-free individuals; N2: normal samples cancer patients; MSI: microsatellite instability; MSS: microsatellite stability.

# References for supplementary information

1. Frommer M, McDonald LE, Millar DS, Collis CM, Watt F, Grigg GW, Molloy PL, Paul CL: **A genomic sequencing protocol that yields a positive display of 5-methylcytosine residues in individual DNA strands.** *Proc Natl Acad Sci U S A* 1992, **89:**1827-1831.

2. Clark SJ, Harrison J, Paul CL, Frommer M: **High sensitivity mapping of methylated cytosines.** *Nucleic Acids Res* 1994, **22:**2990-2997.

3. Grunau C, Clark SJ, Rosenthal A: **Bisulfite genomic sequencing: systematic investigation of critical experimental parameters.** *Nucleic Acids Res* 2001, **29:**E65.

4. Lind GE, Kleivi K, Meling GI, Teixeira MR, Thiis-Evensen E, Rognum TO, Lothe RA: **ADAMTS1, CRABP1, and NR3C1 identified as epigenetically deregulated genes in colorectal tumorigenesis.** *Cell Oncol* 2006, **28:**259-272.

5. **Ensembl Homepage**

6. Herman JG, Graff JR, Myöhänen S, Nelkin BD, Baylin SB: **Methylation-specific PCR: a novel PCR assay for methylation status of CpG islands.** *Proc Natl Acad Sci U S A* 1996, **93:**9821-9826.

7. Alaminos M, Davalos V, Cheung NK, Gerald WL, Esteller M: **Clustering of gene hypermethylation associated with clinical risk groups in neuroblastoma.** *J Natl Cancer Inst* 2004, **96:**1208-1219.

8. Lind GE, Ahlquist T, Lothe RA: **DNA hypermethylation of MAL: a promising diagnostic biomarker for colorectal tumors.** *Gastroenterology* 2007, **132:**1631-1632.

9. Esteller M, Hamilton SR, Burger PC, Baylin SB, Herman JG: **Inactivation of the DNA repair gene O6-methylguanine-DNA methyltransferase by promoter hypermethylation is a common event in primary human neoplasia.** *Cancer Res* 1999, **59:**793-797.

10. Lind GE, Thorstensen L, Løvig T, Meling GI, Hamelin R, Rognum TO, Esteller M, Lothe RA: **A CpG island hypermethylation profile of primary colorectal carcinomas and colon cancer cell lines.** *Mol Cancer* 2004, **3:28**.

11. Garcia JM, Silva J, Pena C, Garcia V, Rodriguez R, Cruz MA, Cantos B, Provencio M, Espana P, Bonilla F: **Promoter methylation of the PTEN gene is a common molecular change in breast cancer.** *Genes Chromosomes Cancer* 2004, **41:**117-124.

12. Goel A, Arnold CN, Tassone P, Chang DK, Niedzwiecki D, Dowell JM, Wasserman L, Compton C, Mayer RJ, Bertagnolli MM et al.: **Epigenetic inactivation of RUNX3 in microsatellite unstable sporadic colon cancers.** *Int J Cancer* 2004, **112:**754-759.

13. Krop I, Player A, Tablante A, Taylor-Parker M, Lahti-Domenici J, Fukuoka J, Batra SK, Papadopoulos N, Richards WG, Sugarbaker DJ et al.: **Frequent HIN-1 Promoter Methylation and Lack of Expression in Multiple Human Tumor Types.** *Mol Cancer Res* 2004, **2:**489-494.

14. Smith-Sørensen B, Lind GE, Skotheim RI, Fosså SD, Fodstad O, Stenwig AE, Jakobsen KS, Lothe RA: **Frequent promoter hypermethylation of the O6-Methylguanine-DNA Methyltransferase (MGMT) gene in testicular cancer.** *Oncogene* 2002, **21:**8878-8884.

15. Zhou XP, Hoang JM, Li YJ, Seruca R, Carneiro F, Sobrinho-Simoes M, Lothe RA, Gleeson CM, Russell SE, Muzeau F et al.: **Determination of the replication error phenotype in human tumors without the requirement for matching normal DNA by analysis of mononucleotide repeat microsatellites.** *Genes Chromosomes Cancer* 1998, **21:**101-107.

16. Diep CB, Thorstensen L, Meling GI, Skovlund E, Rognum TO, Lothe RA: **Genetic tumor markers with prognostic impact in Dukes' stages B and C colorectal cancer patients.** *J Clin Oncol* 2003, **21:**820-829.
