# Supplementary figures and images for: Gene methylation profiles of normal mucosa, and benign and malignant colorectal tumors identify early onset markers
Source: Mol Cancer. 2008 Dec 31;7:94. doi: 10.1186/1476-4598-7-94 (PMC2639620; doi:10.1186/1476-4598-7-94)

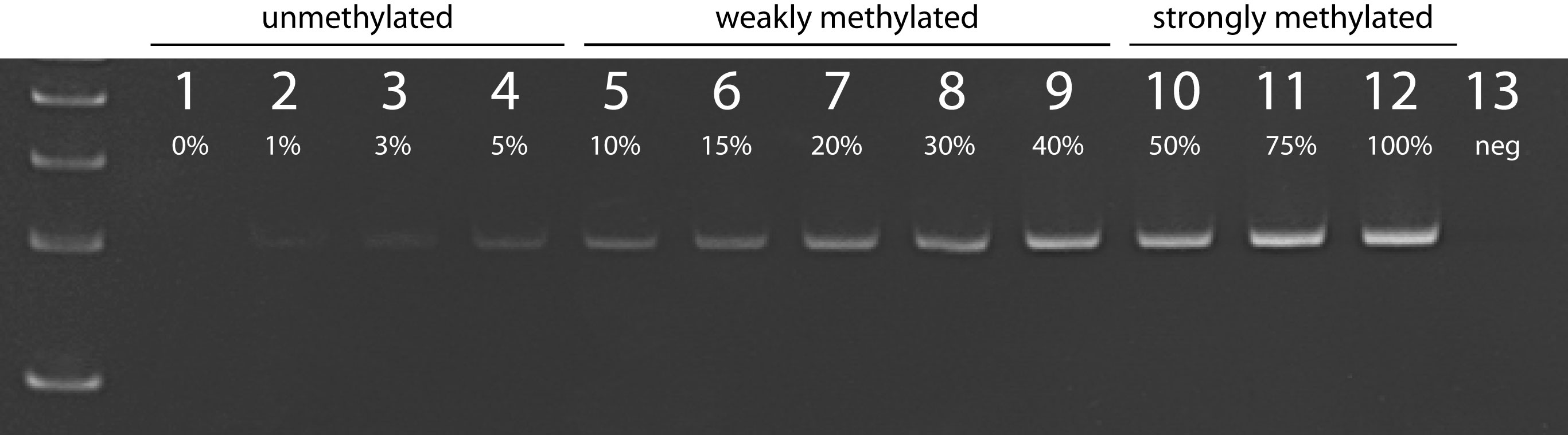

Supplement: Additional file 2 — Titration of methylated DNA template illustrates the scoring thresholds for the methylation-specific polymerase chain reaction. Determination of scoring thresholds is visualized by a titration series of the RUNX3 gene. [file 1476-4598-7-94-S2.jpeg]

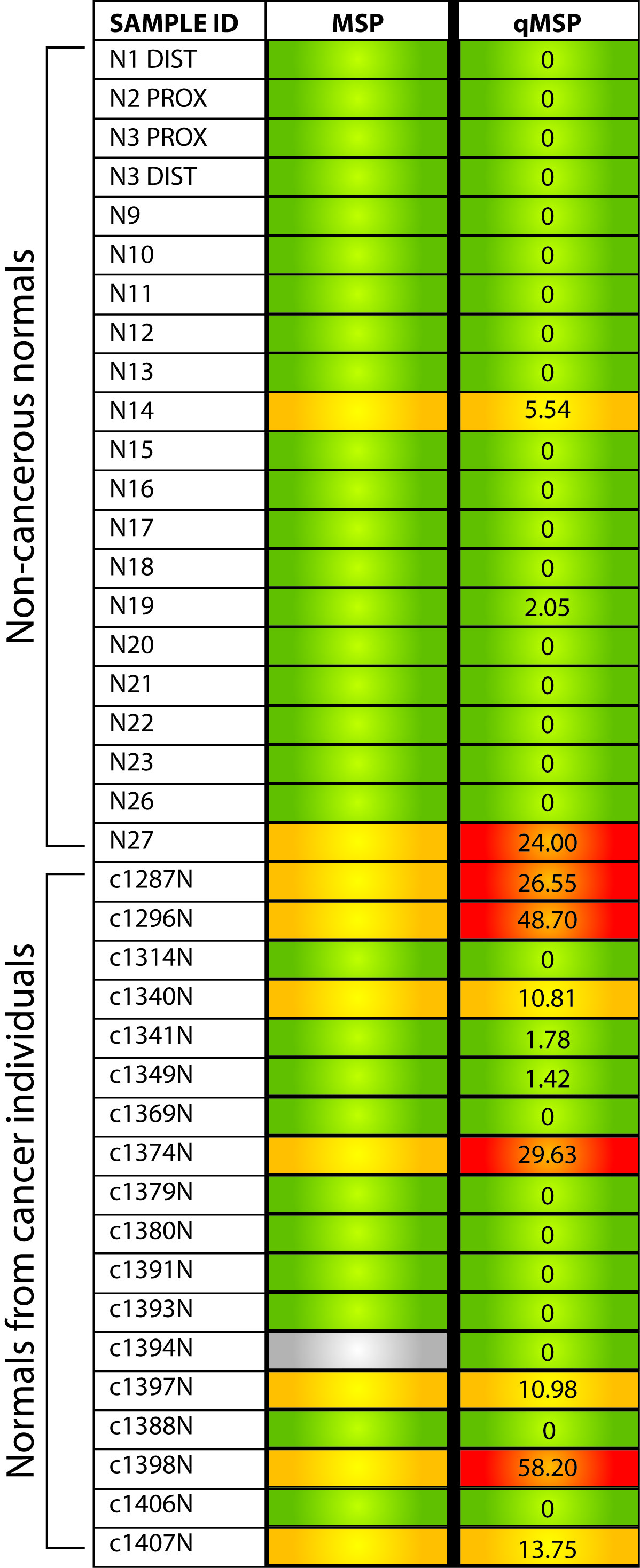

Supplement: Additional file 6 — Comparison between MSP and quantitative MSP in normal mucosa samples. Hypermethylation of was analyzed with both non-quantitative- and quantitative MSP. Here the results from each method are presented. [file 1476-4598-7-94-S6.jpeg]

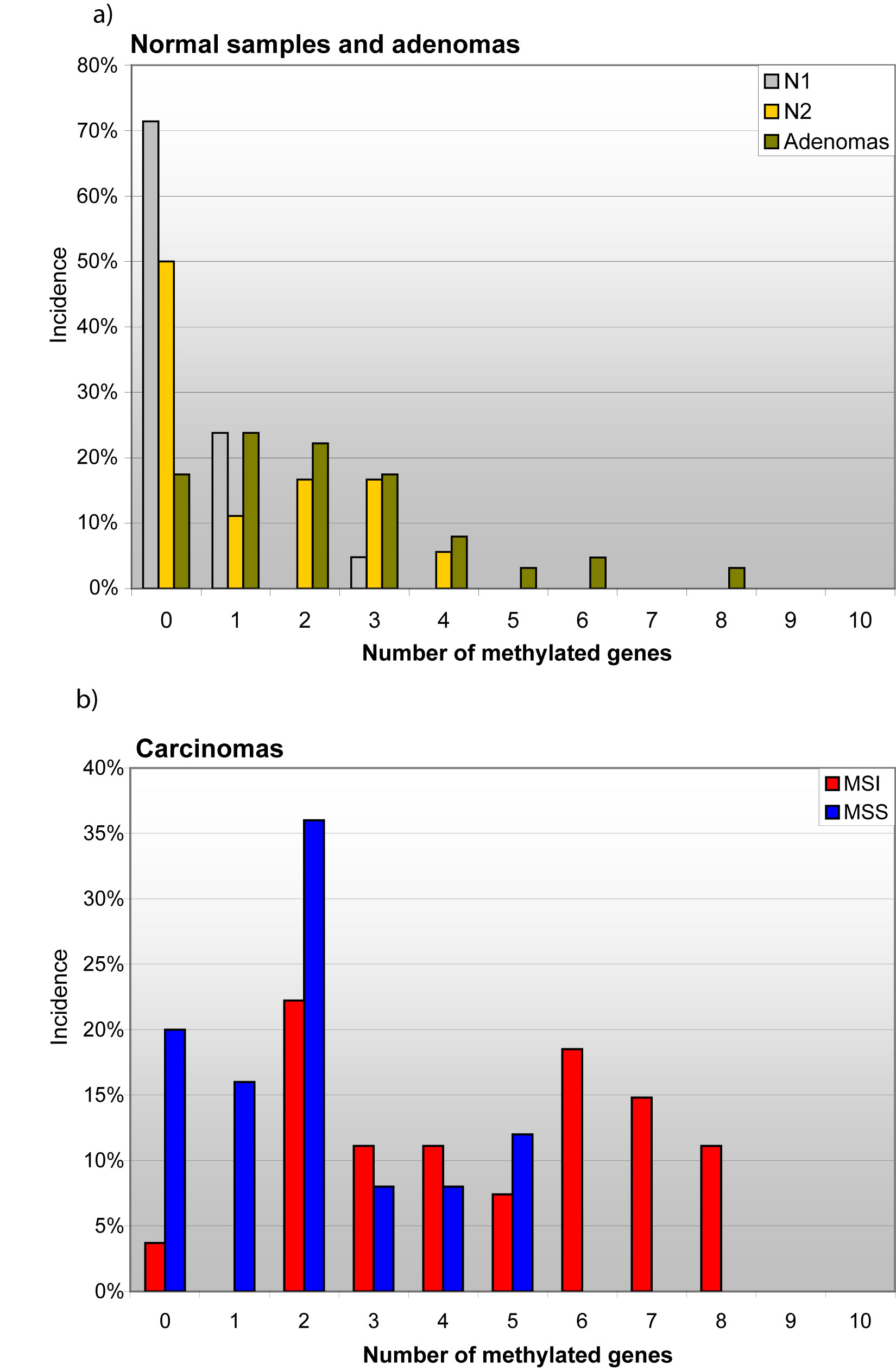

Supplement: Additional file 8 — Widespread methylation among normal colorectal samples, adenomas, and carcinomas. A histogram showing the total number of methylated genes per sample in non-cancerous normal mucosa, normal mucosa taken in distance from a primary tumor, adenomas, and carcinomas stratified according to MSI status. [file 1476-4598-7-94-S8.jpeg]
